# Determinants of higher education teachers’ intention to use technology-based exams

**DOI:** 10.1007/s10639-022-11435-4

**Published:** 2022-11-17

**Authors:** Aron Fink, Christian Spoden, Andreas Frey

**Affiliations:** 1 Department of Educational Psychology, Institute of Psychology, Goethe University Frankfurt, Theodor-W.-Adorno-Platz 6, 60323 Frankfurt, Germany; 2 University of Applied Science Emden/Leer, Emden, Germany

**Keywords:** Technology-based assessment, Computerized adaptive testing, Higher education, Technology acceptance

## Abstract

The replacement of existing technology or the introduction of novel technology into the day-to-day routines of higher education institutions is not a trivial task. Currently, many higher education institutions are faced with the challenge of replacing existing procedures for administering written exams with e-exams. To guide this process, this paper proposes the novel technology-based exams acceptance model (TEAM) and empirically evaluates its model structure and usefulness from the perspective of higher education teachers. The model can be used to guide the transition from paper-based exams to e-exams and the implementation of innovative (e.g., adaptive) e-exam formats. The model includes perceived usefulness, computer self-efficacy, computer anxiety, prior experience, facilitating conditions, and subjective norm as predictors of the behavioral intention to use e-exams. To test the model empirically, the responses of 992 teachers at 63 German universities to a standardized online questionnaire were analyzed using structural equation modeling. The model fit was acceptable. With 77% (conventional e-exams) and 82% (adaptive e-exams), a large proportion of the variance of the intention to use these types of exams was explained. With TEAM, a highly predictive model for explaining the behavioral intention to use e-exams is now available. It offers a theoretical basis that can be used for the successful implementation of e-exams in higher education.

## Introduction

Digital transformation affects nearly all areas of modern life. This also includes higher education. When it comes to testing student performance in terms of written examinations, a shift from paper-based exams to computer-administered exams has begun. Furthermore, the COVID-19 pandemic and the associated forced shift to digital learning in higher education institutions around the globe can be seen as a tipping point for the integration of digital technologies for exam purposes into higher education (St-Onge et al., 2021).

The use of computer-based assessments (CBA) as summative assessment tools in higher education can be subsumed under the term e-exams. In this article, e-exams are defined as timed, summative high-stakes assessments of student performance that use digital devices to run a standardized assessment system and in which responses are submitted and, for the most part, scored electronically (e.g., Fluck, 2019). The use of e-exams has several advantages compared to conventional paper-based exams, such as increased test security, cost and time reduction, automated test assembly and analysis of test responses, rapid or even immediate feedback on results, the possibility to integrate interactive elements and multimedia tools into the assessment process, provision of more authentic tests, and the possibility of automatic record keeping for item analysis (Boevé et al., 2015; Nikou & Economides, 2018a; Rolim & Isaias, 2019; Spoden & Frey, 2021; St-Onge et al., 2021). Furthermore, compared to conventional paper-based testing, using CBA can have positive effects on students’ test-taking motivation, self-efficacy, test perception, and even test performance (Chua & Don, 2013; Gu et al., 2020; Nardi & Ranieri, 2018; Nikou & Economides, 2016; Rolim & Isaias, 2019).

In addition, the use of digital technologies for examination purposes provides the opportunity to integrate state-of-the-art methods from psychometrics and psychological evaluation into the testing practice (see, e.g., Spoden & Frey, 2021). In particular, new methods for item calibration such as the continuous calibration strategy (Born et al., 2019; Fink et al., 2018; Frey & Fink, in press) make it possible to combine conventional e-exams with the modern assessment technology of computerized adaptive testing (CAT; Frey, in press). CAT is a testing mode in which the selection of the items to be presented to the test taker depends on the test taker’s responses to the most recent items administered. Therefore, the tests are tailored to the individual ability level of the test takers. This typically yields substantially higher measurement precision and/or a shorter test length compared to conventional nonadaptive testing (Segall, 2005). Furthermore, conventional nonadaptive tests typically have the problem that they provide the highest measurement precision for test takers of medium ability, while the precision decreases for test takers with high or low test scores (Dolan & Burling, 2017); adaptive e-exams can help to solve this problem by aligning the standard errors across the complete ability range. Thus, adaptive e-exams can provide teachers in higher education with highly reliable measures of student abilities.

However, the implementation of e-exams that leverage current assessment advancements and digital technologies in higher education is not trivial. Rather, several conditions have to be met for this to happen. Research from the area of technology acceptance (e.g., Marangunić & Granić, 2015) provides reference points for the necessary conditions for successful technology integration in general (e.g., Abdullah & Ward, 2016; Al-Emran et al, 2018; Granić & Marangunić, 2019; Scherer & Teo, 2019). Technology acceptance comprises different attitudes regarding technology and beliefs that explain a person’s intentions to use technology, as well as their actual use of technology (Davis, 1989). One model in particular has dominated the research on factors that influence the acceptance and use of technologies: the technology acceptance model (TAM; Davis, 1989). The core assumption of TAM is that perceived usefulness (PU) and perceived ease of use (PEOU) are the central factors that influence a person’s attitude toward and behavioral intentions with regard to technology use.

However, TAM and related models focus on technology use in general and not specifically on e-exams. Therefore, in their current form, they do not cover the circumstances relevant for the implementation of e-exams precisely enough. A specific TAM for e-exams in higher education would be very useful for many higher education institutions, where decisions have to be made that make the successful implementation of e-exams possible. The successful implementation of e-exams in the routine procedures of higher education institutions depends, among others, on the technology acceptance of the stakeholders involved. If they do not accept this assessment type, they are often in positions in which they can prevent its implementation regardless of the advantages.

As previous research on the implementation of e-exams in higher education has primarily focused on the acceptance of e-exams by students (Maqableh et al., 2015; Terzis & Economides, 2011; Terzis et al., 2012; Zheng & Bender, 2019), there is a need for studies that explicitly consider the perspectives of the academic staff on e-exams (Bennett et al., 2017; Brady et al., 2019; Deeley, 2018). Especially the viewpoint of the teaching staff, as they are responsible for the design and integration of e-exams into courses, is a critical factor for the successful implementation of e-exams (Bennett et al., 2017; Brady et al., 2019; Nikou & Economides, 2018a; Paiva et al., 2017). In order to avoid time-consuming and expensive failures during the implementation process, a thorough understanding of the conditions necessary for teachers in higher education to accept e-exams as viable evaluation tools and, therefore, to form a strong intention to use them is exceptionally important. In addition, among the few studies that examined the perspectives of higher education teaching staff on e-exams, even fewer are situated in a clearly defined theoretical framework (Brady et al., 2019).

Against this background, this study aimed to formulate a specific theoretical model on the acceptance of e-exams that makes it possible to predict the behavioral intention of higher education teachers to use e-exams. This model is called the technology-based exams acceptance model (TEAM). It draws from TAM and its extensions (e.g., Terzis & Economides, 2011; Venkatesh et al., 2003). TEAM is intended for use in guiding implementation processes to make them successful. In order to justify such use, empirical data was gathered and statistically analyzed with structural equation modeling to test whether the proposed model structure fits the actual response behavior of higher education teachers. After establishing the model structure, TEAM was used to examine the as yet unanswered question of whether different conditions have to be met before implementing innovative adaptive e-exams compared to the conditions that need to be met before implementing conventional e-exams, which basically mimic paper–pencil exams with computers.

The study had the following four research objectives (ROs):

- RO1: To formulate TEAM.
- RO2: To examine the appropriateness of TEAM for teachers in higher education.
- RO3: To statistically test the theoretically derived direct and indirect effects described by TEAM.
- RO4: To examine whether there are differences with regard to the structure and the path coefficients of TEAM between adaptive e-exams and conventional e-exams.

The text is organized as follows: The next section describes theoretical perspectives on technology acceptance models and the most relevant previous studies on technology acceptance in education. Based on this literature review, hypotheses are derived and TEAM is formulated. The following section covers the methods used to test the hypotheses and to examine the model. Subsequently, the results are presented. Finally, the results are discussed regarding the research objectives, along with practical implications and pathways for future research on TEAM.

### Educational technology acceptance model

TAM is the most frequently used theory in technology acceptance literature in general (e.g., Marangunić & Granić, 2015) and in e-learning acceptance literature in particular (e.g., Abdullah & Ward, 2016; Granić & Marangunić, 2019; Scherer et al., 2019). In the context of educational technologies, numerous studies have explored the applicability of TAM and connected models across a broad range of technologies. Among these are, for instance, mobile learning (Mutambara & Bayaga, 2021; Sánchez-Prieto et al., 2016), digital learning environments (Bauwens et al., 2020; del Barrio-García et al., 2015), learning management systems (Alharbi & Drew, 2014; Cigdem & Topcu, 2015; Fathema et al., 2015; Sánchez & Hueros, 2010), multimedia platforms adapted for learning (Lee & Lehto, 2013), communication and collaboration applications (Maican et al., 2019), virtual reality (Noble et al., 2022), as well as CBA (Maqableh et al., 2015; Terzies & Economides, 2011) or mobile-based assessment (Nikou & Economides, 2017, 2018b).

TAM originates in the theory of reasoned action (Ajzen & Fishbein, 1980). It comprises several variables that directly or indirectly explain the behavioral intention to use technology and the actual use of technology. In the original model, Davis (1989) suggested that three factors influence technology use: perceived ease of use (PEOU), perceived usefulness (PU), and attitude toward using (ATU). PU is defined as a person’s belief about the degree to which using the particular system would enhance their job performance. PEOU is a person’s belief about the degree to which using the particular system would be free of effort (Davis, 1989). Davis (1989) hypothesized that ATU is the main determinant of technology use. PU and PEOU are considered to influence ATU.

Subsequent TAM developments added the behavioral intention (BI) of a person to use a particular system as a new variable that was directly influenced by the PU of the system (Davis et al., 1989). In addition, Davis et al. (1989) argued that there would be cases where an individual could form a strong BI to use the particular system that was perceived as useful without forming any kind of attitude toward using the system and, thus, he removed the ATU construct from the model. In line with this, a large body of studies has underlined the weak role of ATU as a mediator between BI, PU, and PEOU (Davis et al., 1989; Szajna, 1996; Teo, 2009; Venkatesh & Davis, 2000; Wang & Wang, 2009; Yen et al., 2010). Therefore, Venkatesh (2000) considered the simplified version of TAM to be superior to the original model in predicting user acceptance, by including the direct effects of both PU and PEOU on BI.

**Fig. 1:**
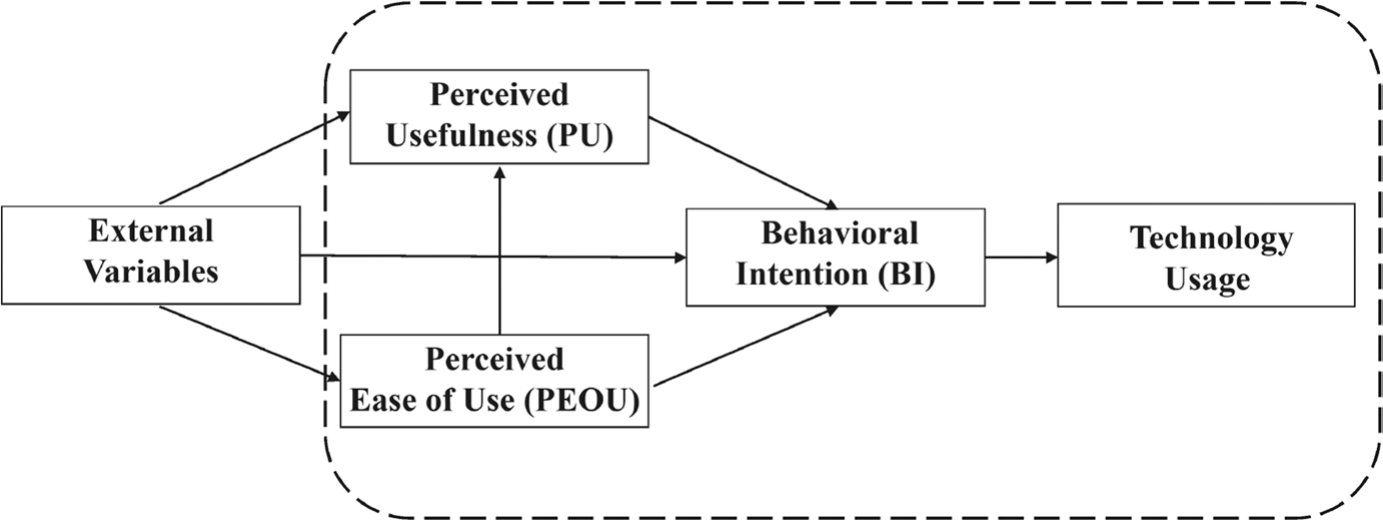
Technology Acceptance Model (TAM)

**Fig. 2:**
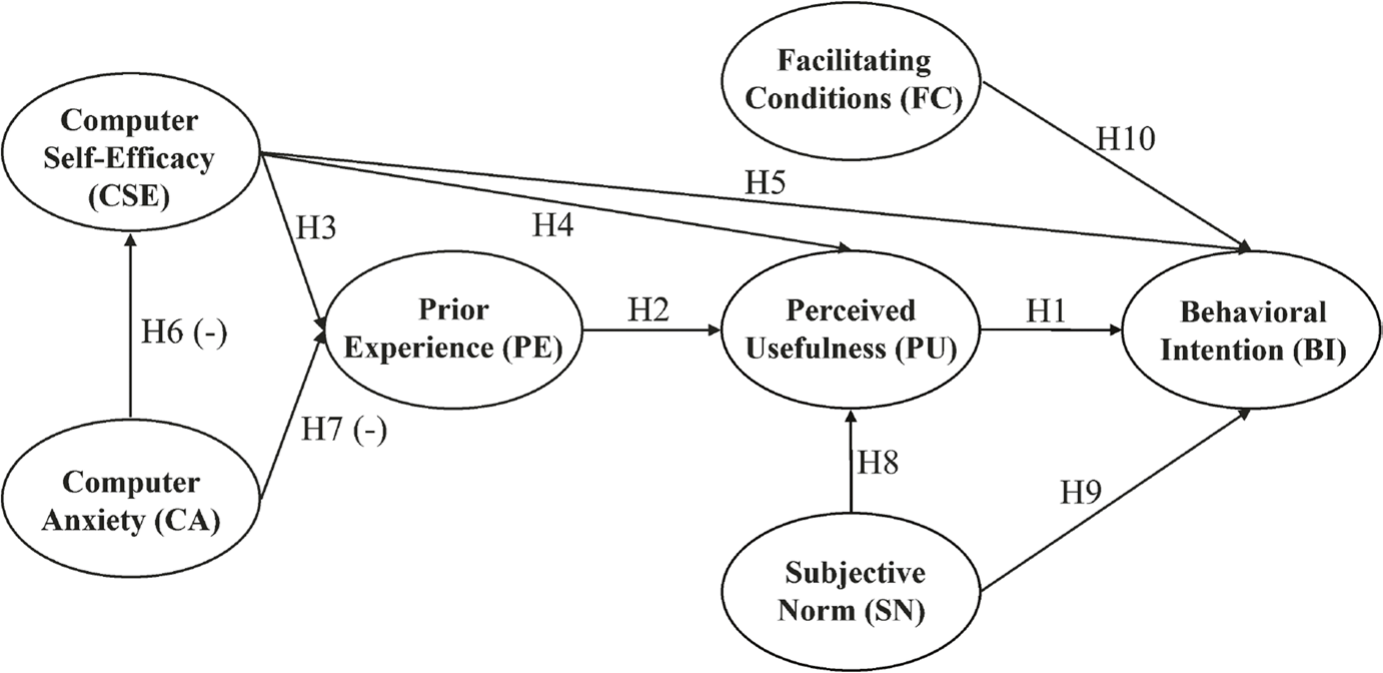
Technology-Based Exams Acceptance Model (TEAM). *Note.* (-) = negative effect. H1–H10 = Hypothesis 1–10

TAM has been modified and extended by different authors over the last decades by having external factors added that explain variation in the TAM core variables PU, PEOU, or BI (Fig. 1; see Marangunić & Granić, 2015 for an overview). This has resulted in a large number of different external factors and several extended TAMs in the research area of e-learning acceptance (Abdullah & Ward, 2016). These external factors represent individual characteristics and beliefs as well as contextual factors. Among others, subjective norm, facilitating conditions, computer self-efficacy, computer anxiety, and prior experience are the most commonly used external factors in the context of e-learning that have been found to be significantly related to the TAM core variables by means of meta-analyses (Abdullah & Ward, 2016; Schepers & Wetzels, 2007; Scherer et al., 2019) or in the systematic review by Granić and Marangunić (2019). These theoretical considerations and empirical findings are presented and discussed in the next section to formulate an empirically testable model of possible factors that influence the intention to use e-exams, both conventional and adaptive, from the perspective of higher education teachers (Fig. 2).

### Hypotheses and model derivation

#### Perceived Usefulness (PU)

PU is one of the core variables in TAM and its extensions. There is solid empirical evidence for a positive effect of PU on teachers’ BI to use educational technologies (e.g., Cigdem & Topcu, 2015; Harris et al., 2016; Lin et al., 2013; Motaghian et al., 2013). In addition, Granić and Marangunić (2019) identified PU as the strongest determinant for the adoption of various learning technologies in their systematic review. We expected to find a similar effect for e-exams. Therefore, the first hypothesis was:

- Hypothesis 1: PU has a positive effect on BI.

#### Prior Experience (PE)

King and He (2006) stated that prior experience is one of the best-studied external factors in the TAM context. Research indicates that individuals with more computer-related experience, such as those who use a computer to write emails or who use word processing software, spreadsheet programs, and others, are more likely to show a higher degree of PU and PEOU with regard to a new e-learning system (Abdullah & Ward, 2016; Lee et al., 2013). As it can be assumed that teachers in higher education often carry out such computer activities in their everyday work, prior experience was more explicitly specified for the higher education setting to comprise the prior use of multimedia and technology for teaching purposes instead of computer-related experience in general. We hypothesized that:

- Hypothesis 2: PE has a positive effect on PU.

#### Computer Self-Efficacy (CSE)

CSE is defined as a person’s belief about the ease with which they ca perform a specific task using a computer (Compeau & Higgins, 1995). CSE can affect the BI to use computers because people who believe that they do not have the ability to use computers will avoid using them (Igbaria & Iivari, 1995; Kwon et al., 2007). Conversely, the higher a person’s CSE is, the higher that person’s use of computers will be (Compeau & Higgins, 1995). Ahmad et al. (2010) showed that this connection also holds with regard to the adoption of e-learning by teachers. In addition, the meta-analysis of teachers’ adoption of e-learning conducted by Scherer et al. (2019) identified CSE as one of the strongest antecedents of PU and PEOU. Conceptually, PEOU and CSE have a lot of similarities. As stated above, PEOU reflects the degree to which a person believes that the system of interest is easy to use, which, in turn, is also reflected by their CSE. These commonalities have been supported by empirical studies, and both constructs are sometimes even measured with similar items (Scherer & Teo, 2019; Scherer et al., 2015). In particular, if a study is not about an existing e-exam system but about the hypothetical use of such a system, PEOU can be regarded as an expression of CSE rather than as the actual ease of use of the corresponding system. Therefore, we integrated CSE rather than PEOU into the model and investigated its impact on PU and BI. In addition, as low CSE is assumed to lead to a lower degree of computer use in general, it can be assumed that teachers in higher education with low CSE use less technology for teaching purposes in general and, therefore, show less PE. We hypothesized that:

- Hypothesis 3: CSE has a positive effect on PE.
- Hypothesis 4: CSE has a positive effect on PU.
- Hypothesis 5: CSE has a positive effect on BI.

#### Computer Anxiety (CA)

CA is defined as the degree of apprehension or even fear an individual feels when using a computer (Venkatesh & Morris, 2000). In this study, CA is regarded as a time-persistent trait that contains both cognitive and affective components (e.g., Morris et al., 1981; Richter et al., 2010). A number of studies have shown that CA is associated with the avoidance or reduced use of e-learning systems (Abdullah & Ward, 2016). A discrepancy between educators’ perceptions of their technological competence and the learning effort they have to put into using computers for teaching purposes can often be perceived as threatening and overwhelming. Thus, the anxiety of a teacher in higher education affects both the extent to which and the way in which they use technology in everyday instructional practice (Al-alak & Alnawas, 2011; Mac Callum et al., 2014). Therefore, it can be assumed that teachers with high CA gather less PE. In addition, the lack of technology use due to CA can be assumed to prevent the development of a high degree of CSE (Lee & Huang, 2014). The next two hypotheses were therefore:

- Hypothesis 6: CA has a negative effect on CSE.
- Hypothesis 7: CA has a negative effect on PE.

#### Subjective Norm (SN)

SN is defined as a person’s perception that most people who are important to them think that they should show the behavior in question (Fishbein & Ajzen, 1975). With regard to e-exam adoption by higher education teachers, SN can be regarded as the extent to which a higher education teacher perceives pressure from members in their environment (e.g., colleagues, students, or the administrative staff) to use e-exam systems. The perception of such pressure increases the likelihood to incorporate positive beliefs regarding an e-exam system into one’s own beliefs system. It also increases the probability to perceive the system as useful and to form a strong BI to use it. Prior research on higher education teachers’ e-learning adoption supports this assumption and identified SN as an important determinant of PU and BI (Cigdem & Topcu, 2015; Garcia & Gomez, 2014; McGill et al., 2011; Motaghian et al., 2013; Nikou & Economides, 2018b; Wang & Wang, 2009). We hypothesized that:

- Hypothesis 8: SN has a positive effect on PU.
- Hypothesis 9: SN has a positive effect on BI.

#### Facilitating Conditions (FC)

FC is defined as a person’s perception of the degree to which organizational and technical resources exist to support the use of a particular technology. FC therefore comprises external determinants of technology adoption. Depending on the system, FC comprises many different aspects and is typically operationalized to include aspects of the environment that are designed to remove barriers to using the technology (Venkatesh et al., 2003). The aspects that are relevant for this study are, especially, the provision of organizational and technical support (e.g., skills training, information and supportive material, administrative support, availability of a designated person to help, etc.) and appropriate technical resources for carrying out e-exams (e.g., hardware, software, intranet). If any of these elements are perceived as missing, a person can avoid forming the intention to use an e-exam system. Conversely, it can be assumed that the more supportive the existing conditions are, the more likely it is that a higher education teacher will intend to use an e-exam system. In line with this, Lin et al. (2013), for example, found FC to have a positive effect on higher education teachers’ BI to use podcasting for e-learning. Thus, we hypothesized:

- Hypothesis 10: FC has a positive effect on the BI.

The complete research model including these hypotheses is presented in Fig. 2.

Because the model is intended to be applicable to both the transition from paper-based exams to e-exams and the implementation of innovative adaptive e-exams, it was further assumed that the effects would be the same for conventional and adaptive e-exams. Thus, the last hypothesis was:

- Hypothesis 11: The above-mentioned effects are invariant between conventional and adaptive e-exams.

## Materials and methods

### Participants

To test the formulated hypotheses, a nationwide (Germany), cross-disciplinary, and cross-institution online study, which addressed the staff involved in teaching, was conducted. The sample was acquired via email by contacting the secretaries of every institute of at least two universities and two universities of applied science per federal state and requesting them to forward the questionnaire invitation to the teaching staff of the institute. Participation was voluntary. The overall sample comprised *N* = 1,000 higher education teachers but eight participants were excluded from the analysis due to missing data on almost all items. Thus, the final sample comprised *N* = 992 (sex: 38% female; age: *M* = 44.29, *SD* = 11.87) higher education teachers distributed across all 16 federal states of Germany, 63 higher education institutions, and 35 disciplines (see Table 2 for the distribution of the sample across disciplines). The participants were randomly assigned to one of two groups. Group 1 comprised *N*_*ex*_ = 494 higher education teachers who responded to a questionnaire on conventional e-exams and Group 2 comprised *N*_*ad*_ = 498 higher education teachers who responded to a questionnaire on adaptive e-exams (see Table 1 for demographic information).

**Table 1:** Demographics of the two Subsamples

	Group	
Variable	Group 1 (e-exams)	Group 2 (adaptive e-exams)
*N*	494	498
Sex	female: 38.9%	female: 37.2%
Average age (*SD*)	44.30 (11.95)	44.29 (11.80)
Highest academic degree		
• Post-doctoral degree	23.3%	22.9%
• Doctoral degree	44.9%	47.2%
• Master’s degree	30.2%	27.7%
• Bachelor’s degree	0.4%	1.0%
• Other	1.2%	1.2%
Already used e-exams for summative assessments	15.6%	15.1%

### Measures and instruments

Along with some demographic questions, both online questionnaires comprised multiple-item scales, ensuring a reliable measurement of the above-mentioned constructs. A mixture of existing scales and scales developed anew by the authors was used. For CSE and PE, corresponding scales from the German version of the teacher questionnaire used in the International Computer and Information Literacy Study 2013 (ICILS; Gerick et al., 2018) were used. As the original items were developed to be used on secondary school teachers, the wording of the items was adapted to the context of higher education. The changes made in the German version of the items is given in Appendix A (Table 5). For CSE, the participants had to rate different computer-related tasks (e. g. *collaborating with others using shared resources such as Google Docs®*) on a three-point Likert scale with the response categories *I do not think I could do this*, *I could work out how to do this*, and *I know how to do this.* For PE, the participants were asked to indicate how often they used different information and communication technology (ICT) tools (e.g. *digital learning games*, *communication software*) when teaching on a four-point Likert scale with the response categories *never*, *in some lessons*, *in most lessons*, and *in every or almost every lesson*. The instrument for CA was adapted from Richter et al. (INCOBI-R; 2010). The items (e.g. *When my computer crashes, I panic*) had a five-point Likert scale ranging from *strongly disagree* to *strongly agree* with the middle category labeled *neutral*. The remaining scales for PU, SN, FC, and BI were developed by the authors. Prior to their use in the study presented here, these scales were trialed and optimized (*N*_*pre*_ = 109 teachers from a German university; Klösel, 2018). The items of these scales had a four-point Likert scale, ranging from *totally disagree* to *totally agree*. The item wording of the developed scales with their English translation is given in Appendix A (Table 6). For each scale used in the questionnaire, higher response categories and therefore also higher scale values reflect higher expressions of the respective construct measured. The respondents had to provide an answer to every item. As it could not be assumed that each of the participants had a deeper understanding of the concepts of e-exams and adaptive e-exams, both terms were explained in the online questionnaires and the main advantages and disadvantages were mentioned.

### Data collection procedures

At the beginning of the study, the participants were provided with information about the study and were asked for consent for their data to be used in the study. Afterwards, each participant was asked to answer an online questionnaire. Two versions of the online questionnaires were used; the first one focused on conventional e-exams and the second one on adaptive e-exams. The online questionnaire versions were assigned randomly to the participants. As mentioned above, the participants were required to complete all items in the questionnaire and were not allowed to skip items. They generally completed the questionnaire within 20 min.

### Data analysis procedures

Once the data were gathered, the explanatory model was tested by means of multigroup structural equation modeling (MG-SEM) in Mplus 8.8 (Muthén & Muthén, 2022). Weighted least square mean and variance adjusted (WLSMV) estimation was used to model the ordinal data. This estimation method utilizes polychoric correlations to estimate bivariate relationships between ordinal indicators. We used the Mplus TYPE = COMPLEX procedure, which adjusts model fit statistics and standard errors for error dependencies caused by the clustered structure of the data. The higher education teachers in the sample are nested in disciplines, which in turn are nested in higher education institutions. Therefore, we used disciplines within institutions as the clusters. Because the clusters can become too small, for the disciplines, the main categories were used (printed in bold in Table 2). As participants were required to complete all items except the items asking for demographic information, only a few items had missing responses (due to test aborts) and, in those cases, the missing rates were very low (< 0.5% per item). The few missing responses were assumed to be missing completely at random and were treated by pairwise deletion as implemented in Mplus when using WLSMV. As a first step, we estimated the measurement model (simple structure with correlated factors, see Appendix B) in both groups (Group 1 [e-exams], Group 2 [adaptive e-exams]) via multigroup confirmatory factor analysis (MG-CFA), and we examined the loading pattern. Additionally, when testing the hypotheses and comparing the groups, we conducted a measurement invariance analysis to examine whether the constructs had been measured in a directly comparable manner in the two groups. In a second step, we conducted the full MG-SEM and inspected model fit (RO1). In this analysis, we estimated the model shown in Fig. 2. Afterwards, the statistical significance of the proposed relations was examined separately for each group (Hypotheses 1–10). Hypothesis 11 was tested by means of Wald tests that compared latent means and path coefficients between the two groups.

**Table 2:** Distribution of the sample across disciplines

	Group 1 (e-exams)		Group 2 (adaptive e-exams)	
Discipline	N	%	N	%
**Natural sciences**	**143**	**28.9**	**136**	**27.5**
Mathematics	16	3.2	14	2.8
Computer science	41	8.3	44	8.9
Physics and Astronomy	27	5.5	28	5.7
Cheminstry	18	3.6	10	2.0
Environmental science	13	2.6	14	2.8
Biology	15	3.0	17	3.4
Others	13	2.6	9	1.8
**Engineering and technology**	**97**	**19.6**	**88**	**17.8**
Civil engineering	19	3.8	14	2.8
Electrical engineering	15	3.0	26	5.3
Mechanical enginieering	42	8.5	32	6.5
Materials engineering	-	-	-	-
Medical engineering	3	0.6	2	0.4
Environmental engineering	4	0.8	1	0.2
Biotechnology	1	0.2	3	0.6
Nanotechnology	1	0.2	-	-
Others	13	2.6	10	2.0
**Medicine and health sciences**	**24**	**4.9**	**30**	**6.1**
Medicine	18	3.6	22	4.5
Health sciences	4	0.8	5	1.0
Others	2	0.4	3	0.6
**Agriculture and forestry**	**9**	**1.8**	**12**	**2.4**
Agriculture, forestry and fisheries	7	1.4	10	2.0
Veterinary medicine	-	-	1	0.2
Others	2	0.4	1	0.2
**Social and behavioral sciences**	**104**	**21.1**	**131**	**26.5**
Psychology	8	1.6	22	4.5
Economics	34	6.9	56	11.3
Educational sciences	27	5.5	20	4.0
Sociology	8	1.6	7	1.4
Law	8	1.6	9	1.8
Political sciences	3	0.6	2	0.4
Social and economic geography	1	0.2	1	0.2
Media and communication sciences	2	0.4	2	0.4
Others	13	2.6	12	2.4
**Humanities**	**99**	**20.0**	**82**	**16.6**
History and archaeology	13	2.6	7	1.4
Linguistics and literatur	59	11.9	47	9.5
Philosophy, ethics and religious studies	15	3.0	15	3.0
Art, music, theater and media studies	2	0.4	4	0.8
Others	10	2.0	9	1.8

## Results

### Descriptive results and measurement model

Table 3 shows latent correlations as well as Green and Yang’s (2009) variation of coefficient ω as a measure of reliability for categorical data. The ω exceeded the suggested rule of thumb of 0.70 for all scales, so that the reliability of all scales can be regarded as acceptable or better. The standardized factor loadings of the items ranged from 0.646 to 0.949 for e-exams and from 0.651 to 0.948 for adaptive e-exams (see Appendix B for a detailed presentation of the results of the measurement model). The fit of the measurement model (see fit measures of the configural model in Table 3) can be regarded as acceptable.

**Table 3:** Reliability, and latent correlations

Group	Factor	ω	BI	PU	PE	CSE	CA	SN	FC
Group 1 (e-exams)	BI	0.881	1.00	-	-	-	-	-	-
	PU	0.874	**0.867**	1.00	-	-	-	-	-
	PE	0.858	**0.297**	**0.271**	1.00	-	-	-	-
	CSE^a^	0.853	**0.185**	**0.210**	**0.467**	1.00	-	-	-
	CA	0.871	-0.003	-0.042	**-0.121**	**-0.492**	1.00	-	-
	SN	0.717	**0.630**	**0.634**	**0.315**	**0.230**	0.054	1.00	-
	FC	0.873	**0.340**	**0.275**	**0.212**	**0.220**	0.016	**0.700**	1.00
Group 2 (adaptive e-exams)	BI	0.916	1.00	-	-	-	-	-	-
	PU	0.940	**0.900**	1.00	-	-	-	-	-
	PE	0.809	**0.243**	**0.278**	1.00	-	-	-	-
	CSE^a^	0.835	**0.183**	**0.188**	**0.486**	1.00	-	-	-
	CA	0.832	0.072	0.067	**-0.118**	**-0.532**	1.00	-	-
	SN	0.722	**0.227**	**0.281**	**0.221**	0.092	0.053	1.00	-
	FC	0.902	**0.098**	**0.114**	**0.225**	0.094	0.018	**0.625**	1.00

Correlation coefficients significantly different from 0 (*p* ≤ 0.05) are printed in bold. ω = Green and Yang’s (2009) variation of coefficient ω; BI = Behavioral intention to use; PU = Perceived usefulness, PE = Prior experience; CSE = Computer self-efficacy; CA = Computer anxiety; SN = Subjective norm; FC = Facilitating conditions

^a^ Six items were removed due to limited variance

### Measurement invariance analysis

In order to test the measurement invariance of the proposed measurement model across the two groups, we used the four-step approach to test measurement invariance (e.g., van de Schoot et al., 2012). This includes analyzing (1) configural invariance (noninvariance model), (2) metric invariance (invariant factor loadings across groups), (3) scalar invariance (invariant factor loadings and thresholds across groups), and (4) residual invariance (invariant factor loadings, thresholds, and residual variances across groups). Measurement invariance is usually determined by testing whether the difference in the global model fit between the compared groups, $\Delta {\upchi }^{2}$, differs from zero to a statistically significant extent (Byrne et al., 1989). However, because $\Delta {\upchi }^{2}$ is sensitive to sample size, Chen (2007) recommends using the change in alternative globalmodel fit indices as a criterion as well. Chen suggests a criterion of a -0.01 maximum change in the comparative fit index (CFI), together with changes in the root mean squared error of approximation (RMSEA) 0.015 and the standardized root mean square residual (SRMR) of 0.030, for metric invariance or 0.015 for scalar or residual invariance.

Table 4 shows the results of the measurement invariance tests: the residual invariance of the factors across the two groups can be regarded as established, with all changes in alternative global model fit indices smaller than the criteria mentioned above.

**Table 4:** Result of measurement invariance tests for the two analyzed groups

Model	χ^2^ (*df*)	CFI	RMSEA (90% CI)	SRMR	Δχ^2^ (Δ*df*)	ΔCFI	ΔRMSEA	ΔSRMR
configural	5409.210 (3154)	0.944	0.034 (0.032/0.035)	0.091	-	-	-	-
metric	5599.761 (3205)	0.942	0.035 (0.032/0.036)	0.092	190.55* (51)	-0.001	0.001	0.001
scalar	5877.517 (3366)	0.941	0.036 (0.034/0.038)	0.092	255.78* (161)	-0.002	0.001	0.001
residual	5916.088 (3380)	0.937	040 (0.038/0.041)	0.093	38.37* (14)	-0.004	0.004	0.001

CFI = Comparative Fit Index; RMSEA = Root Mean Square Error of Approximation; SRMR = Standardized Root Mean Square Residual

* *p* ≤ 0.05

### Overall model fit

Following the investigation of the measurement model and the measurement invariance analysis, the full MG-SEM was estimated. As a first step, the overall global model fit of the research model was evaluated. The results indicated that the research model had an acceptable fit: χ^2^ = 5787.957, *df* = 3322, CFI = 0.945, TLI = 0.946, SRMR = 0.094, RSMEA = 0.039 (90% CI [0.038, 0.041]). In both groups, a very large proportion of the BI was explained with the suggested model (Group 1 [e-exams]: 77.4%; Group 2 [adaptive e-exams]: 82.4%). Thus, TEAM can be regarded as robust and as being able to explain higher education teachers’ intention to use (adaptive) e-exams well.

### Hypotheses testing

**Fig. 3:**
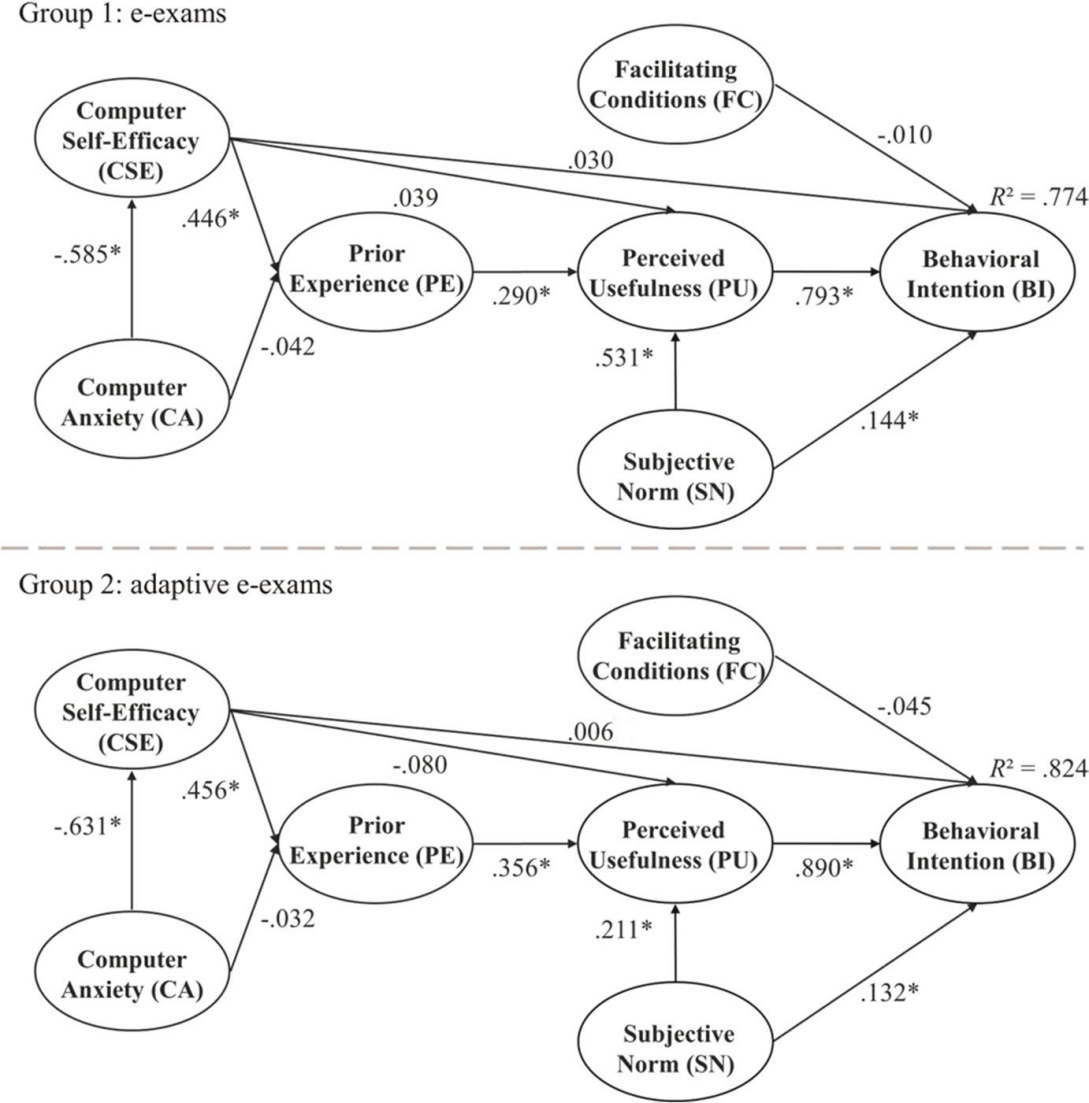
Results for the MG-SEM analysis. *Note.* * *p* ≤ 0.05, standardized path coefficients

As a second step, and addressing RO2, the proposed relations were statistically tested separately for each group. Figure 3 summarizes the results for the hypotheses. In both groups, PU had a strong positive effect on BI (Hypothesis 1; Group 1 [e-exams]: *p* < 0.001; Group 2 [adaptive e-exams]: *p* < 0.001). PE had a positive effect on PU for conventional e-exams as well as for adaptive e-exams (Hypothesis 2; Group 1 [e-exams]: *p* < 0.001; Group 2 [adaptive e-exams]: *p* < 0.001). Regarding CSE, as hypothesized, in both groups, a direct positive effect on PE (Hypothesis 3; Group 1 [e-exams]: *p* < 0.001; Group 2 [adaptive e- exams]: *p* < 0.001) was found but there were no direct effects on PU (Hypothesis 4; Group 1 [e-exams]: *p* = 0.532; Group 2 [adaptive e-exams]: *p* = 0.249). In both groups CSE had no effect on BI (Hypothesis 5; Group 1 [e-exams]: *p* = 0.290; Group 2 [adaptive e-exams]: *p* = 0.795). In addition, in both groups, CSE had a significant indirect effect on PU that was mediated by PE (Group 1 [e- exams]: *β*_ind_ = 0.204, *p* < 0.001; Group 2 [adaptive e-exams]: *β*_ind_ = 0.362, *p* < 0.001) but it did not have an indirect effect on BI via PU (Group 1 [e-exams]: *β*_ind_ = 0.054, *p* = 0.534; Group 2 [adaptive e-exams]: *β*_ind_ = 0.015, *p* = 0.249). CA was found to have a medium direct negative effect on CSE in both groups (Hypothesis 6 Group 1 [e-exams]: *p* < 0.001; Group 2 [adaptive e-exams]: *p* < 0.001). There was no direct effect of CA on PE (Hypothesis 7; Group 1 [e-exams]: *p* = 0.346; Group 2 [adaptive e-exams]: *p* = 0.150), but there was an indirect effect that was mediated by CSE (Group 1 [e- exams]: *β*_ind_ = -0.261, *p* < 0.001; Group 2 [adaptive e-exams]: *β*_ind_ = -0.288, *p* < 0.001). As hypothesized, SN had a direct effect on PU (Hypothesis 8; Group 1 [e-exams]: *p* < 0.001; Group 2 [adaptive e-exams]: *p* < 0.001) as well as on BI (Hypothesis 9; Group 1 [e-exams]: *p* = 0.002; Group 2 [adaptive e-exams]: *p* = 0.015). Next to the direct effect, SN had a significant indirect effect on BI that was mediated by PU in both groups (Group 1: [e-exams]: *β*_ind_ = 0.421, *p* < 0.001; Group 2 [adaptive e-exams]: *β*_ind_ = 0.188, *p* < 0.001). Finally, FC did not have an effect on BI (Hypothesis 10; Group 1 [e-exams]: *p* = 0.808; Group 2 [adaptive e-exams]: *p* = 0.113). In total, six out of the 10 hypotheses were supported by the results. A summary of the hypotheses testing can be found in Appendix C.

The results clearly identified PU as the key predictor of BI. In addition, SN had small to medium effects on BI and PU. The effect of FC on BI was negligible. Furthermore, PU was directly affected by PE. CSE was found to have only an indirect effect on PU, which was mediated by PE. CSE, in turn, was negatively related to CA.

### Comparison of conventional and adaptive e-exams

Comparing the two models, and addressing RO3, our results showed significant standardized latent mean differences between the two factors PU (*d* = -0.407, *p* < 0.001) and BI (*d* = -0.234, *p* = 0.004), with higher values obtained for Group 1 (e-exams) than for Group 2 (adaptive e-exams). There were no significant differences in the other latent means between the two groups. Looking at the path coefficients, the results showed that in Group 1 (e-exams), SN had a significantly larger effect on PU ($\mathrm{\Delta \beta }$ = 0.320; *p* < 0.001) and on BI ($\mathrm{\Delta \beta }$ = 0.012; *p* = 0.008) than in Group 2 (adaptive e-exams). Apart from this, PU had a significantly larger effect on BI ($\mathrm{\Delta \beta }$ = 0.097; *p* < 0.001) in Group 2 (adaptive e-exams). The remaining standardized path coefficients did not differ significantly between the two groups. In addition, the intention to use adaptive e-exams was more strongly predicted (*R*^2^ = 82.4%) than the intention to use conventional e-exams (*R*^2^ = 77.4%). Therefore, Hypothesis 11 was only partially supported by our results.

## Discussion

Although e-exams have several advantages compared to conventional paper-based exams, problems in the implementation of e-exams can easily become time-consuming and costly for both higher education institutions and teachers. In the current climate of a fundamental shift towards e-exams in more and more higher education institutions, research is urgently needed that provides a profound understanding of the very specific conditions that must be fulfilled to facilitate the implementation of e-exams by teachers in higher education (Bennett et al., 2017; Brady et al., 2019; Deeley, 2018). This is particularly important for the implementation of adaptive e-exams, which offer major advantages in psychometric quality such as substantially improved measurement efficiency, individualization, an extension of the performance bandwidth that can be measured, and an alignment of measurement precision across students. However, the implementation of adaptive e-exams has not yet been covered by previous research specific to higher education. This study makes three contributions to this area. First, with TEAM, it proposes a theoretical model that makes it possible to predict the intention of higher education teachers to use e-exams (RO1). Second, it provides empirical evidence for the appropriateness of the suggested model’s structure, including the hypothesized effects and the applicability for both conventional e-exams and adaptive e-exams. Third, it can be applied within change processes at higher education institutions to guide successful implementation processes of e-exams and adaptive e-exams.

Specifically, RO2 aimed to test the general capacity of the proposed model to explain higher education teachers’ intention to use (adaptive) e-exams through its factors by finding evidence in terms of model fit. The model was supported by the data. Thus, TEAM can be regarded as a reliable theoretical basis for explaining higher education teachers’ BI to use conventional and adaptive e-exams. With 77% (e-exams) and 82% (adaptive e-exams), large proportions of the variance of BI were explained with the suggested model structure. Therefore, it can be expected that predictions made with TEAM will come very close to the results that can actually be observed. Regarding the influence of the individual factors (RO3), the study resulted in six conclusions:

1. The PU of conventional and adaptive e-exams was the key predictor of the BI to use them.
2. PE in the sense of digital media use in courses led to a higher degree of PU of new educational technologies such as e-exams.
3. CSE did not have a direct effect but did have an indirect effect on PU, which was mediated by PE.
4. High CA led to a lower degree of CSE and, therefore, indirectly and negatively influenced digital media use in courses (PE).
5. The SN resulting from professional social environments played an important role in influencing the BI to use conventional and adaptive e-exams.
6. FC, such as supportive organizational and technical resources, did not have an effect on the BI to use conventional and adaptive e-exams.

RO4 was to investigate whether the results would differ between conventional and adaptive e-exams. The results revealed only few differences between the two groups. SN had a positive effect on PU in both groups. However, the effect was more than twice as high for conventional e-exams than for adaptive e-exams. This could be explained by the fact that up to the present day, with one exception (see Spoden et al., 2022), no German university already used adaptive e-exams. Thus, it must also be assumed that the perceived subjective pressure to use adaptive e-exams cannot have the same effect as the perceived subjective pressure on using conventional e-exams, which are already being used at several German universities. However, this assumption cannot be proved with the data at hand. Future studies could investigate this in more depth, for example, by comparing higher education institutions where adaptive e-exams are used with those where this is not the case.

In addition, the effect of PU on BI was stronger for adaptive e-exams. This could have resulted from the lower effects of SN on BI and PU. Thus, less systematic variance was bound by SN, which could have led to the stronger effect of PU on BI. Moreover, CSE had an effect on the BI to use adaptive e-exams only. Such innovative CBA formats also require the test administrators to have higher technical skills to enable them to follow the principles of CAT. Therefore, it could be assumed that a higher degree of CSE is necessary in order to form a strong BI to use such formats, which results in a positive effect of CSE on BI.

### Outlook

The suggested model proved to be capable of explaining the intention of higher education teachers to use (adaptive) e-exams with six interrelated variables. This intention will often directly translate into behavior. However, high BI does not guarantee subsequent behavior, as has been discussed in the TAM-based research (e.g., Liu et al., 2019; Nistor, 2014; Scherer et al., 2020; Wu & Du, 2012). Therefore, future studies should examine whether a strong BI leads to the actual use of e-exam systems. Furthermore, to take into account the complexities of turning intentions into actual behavior, the moderating effects of contextual and social factors on the intention-behavior link should be considered.

TEAM focuses on e-exam acceptance by higher education teachers. The model does not specify which types of professional knowledge about teaching and learning with technology higher education teachers must have in order to integrate technology into the assessment process in a meaningful way. The technological pedagogical content knowledge (TPACK) framework defines the different interrelated knowledge domains necessary for the educationally useful integration of technology into teaching and learning processes (Mishra & Koehler, 2006). Previous studies have shown that TPACK and educational technology acceptance are interrelated (e.g., Hsu, 2016; Mei et al., 2018). Considering this, it would be interesting for future studies to investigate the connections between TPACK and TEAM in order to get an even deeper understanding of the underlying processes of higher education teachers’ e-exam acceptance.

Finally, it is likely that some disciplines are more willing to use digital technologies for teaching and testing than others. Therefore, results were corrected for possible error dependencies due to the clustered data structure (higher education teachers nested within disciplines within higher education institutions). Note that the sample sizes per cluster were too small to draw valid conclusions when comparing different disciplines. Future studies may take this up by drawing a sample which makes discipline-specific analyses possible.

## Conclusions

With TEAM, an empirically investigated, highly predictive model for explaining BI is now available. This model offers a sound theoretical basis that can be used to optimize the implementation of e-exams. A promising result of this study is that the higher education teachers in our sample did not express a strong need for expensive infrastructural changes (which would be reflected in stronger effects of FC) in order for them to form a strong BI. Rather, according to the study results, the goals of the implementation process should be a) to promote CSE, for example, by means of academic instruction and training; b) to encourage teachers to try out different kinds of digital media in their courses in order for them to become familiar with them and, thus, to gather experience in using technology for teaching purposes in general; and c) to promote e-exams through an appropriate communication strategy and, therefore, increase the perceived SN. These goals seem achievable as it can be assumed that the COVID-19 pandemic and the related shift to online teaching and learning has forced many countries to vigorously pursue goals a) and b) (e.g., St-Onge et al., 2021), and goal c), in turn, can be supported by already existing structures in higher education institutions. The scales published in this article can also be used to evaluate the effectiveness of interventions that aim to reach these goals.

This study determines the conditions necessary for a successful implementation of e-exams as high-stakes assessments at higher education institutions and it offers the essential building blocks required for a goal-oriented and theory-based implementation of e-exams.

## Data Availability

The datasets and materials used and analysed during the current study are available from the corresponding author on reasonable request.

## Appendix A

**Table 5:** Adapted wording for the German versions of the scales used from ICILS 2013 (Translated into English by the Authors)

Original Wording	Adapted Wording
Schülerinnen und Schüler [secondary school students]	Studentinnen und Studenten [students in higher education]
Unterricht [class]	Lehrveranstaltungen [courses]
Unterrichtsstunden [lessons]	Sitzungen [sessions]
Schuljahr [school year]	Semester [semester]

ICILS 2013 = International Computer and Information Literacy Study (Gerick et al., 2018)

**Table 6:** Item wording for the four self-developed scales (Translated into English by the Authors)

Variable	Item	Wording
PU	01	E-Klausuren haben das Potential meine Arbeit zu erleichtern. [E-exams have the potential to make my work easier.]
	02	E-Klausuren haben das Potential meine Effektivität zu steigern. [E-exams have the potential to increase my effectiveness.]
	03	E-Klausuren sind ein guter Weg, um die Prüfungsbelastung zu reduzieren. [E-exams are a good way to reduce the working load of examinations.]
	04	E-Klausuren erhöhen die Qualität von Hochschulklausuren. [E-exams increase the quality of university exams.]
	05	E-Klausuren erleichtern den Prüfungsvorgang. [E-exams facilitate the examination process.]
	06	E-Klausuren ermöglichen eine fairere Bewertung im Vergleich zu papierbasierten Klausuren. [E-exams make fairer evaluations possible compared to paper-based exams.]
	07	E-Klausuren erscheinen mir nützlich. [E-exams seem to be useful.]
	08	Die Nutzung von E-Klausuren eröffnet im Vergleich zu papierbasierten Klausuren bessere Möglichkeiten zur Überprüfung der Lernziele von Lehrveranstaltungen. [Compared to paper-based exams, the use of e-exams opens up better possibilities to evaluate the learning objectives of courses.]
	09	Durch die Verwendung von E-Klausuren kann ich im Vergleich zu papierbasierten Klausuren Zeit bezüglich der Auswertung und Benotung von Klausuren einsparen. [By using e-exams I can save time in the evaluation and grading of exams compared to paper-based exams.]
SN	01	Ich bin der Meinung, dass die Mehrheit der in der Lehre Tätigen an meiner Hochschule E-Klausuren für eine gute Sache halten. [I think that the majority of the teaching staff at my university considers e-exams to be a good thing.]
	02	Meine Kollegen haben mir bereits dazu geraten, E-Klausuren zu nutzen. [My colleagues have already advised me to use e-exams.]
	03	Meine Hochschule unterstützt die Nutzung neuer Technologien in der Lehre (wie z. B. E-Klausuren-Systeme). [My university supports the use of new technologies in teaching (such as, e.g., e-exam systems).]
	04	Meine Hochschule ist darauf bedacht, stets „up-to-date “ zu sein. [My university strives to be constantly up-to-date.]
	05	Meine Studentinnen und Studenten befürworten den Einsatz von E-Klausuren. [My students support the use of e-exams.]
FC	01	An meiner Hochschule stehen Ansprechpartnerinnen und Ansprechpartner zur Verfügung, die mich bei der Entwicklung und Umsetzung von E-Klausuren unterstützen können. [At my university, there are contact persons who can support me in the development and implementation of e-exams.]
	02	Mir sind Fort-/ und Weiterbildungsmöglichkeiten zum Thema E-Klausuren an meiner Hochschule bekannt. [I am aware of training opportunities on the subject of e-exams at my university.]
	03	Die technischen Ressourcen meiner Hochschule (z. B. Computerpools, Intranet) sind zur Durchführung von E-Klausuren geeignet. [The technical resources at my university (e.g., computer pools, intranet) are suitable for running e-exams.]
	04	Ich weiß, an wen ich mich innerhalb meiner Hochschule bei Fragen oder Problemen in Bezug auf E-Klausuren wenden kann. [I know the contact person at my university for questions or problems regarding e-exams.]
	05	Der Einsatz von E-Klausuren ist bereits in der Prüfungsordnung meiner Hochschule geregelt. [The use of e-exams is already regulated in the examination regulations of my university.]
	06	Meine Hochschule fördert den Einsatz von E-Klausuren durch die Bereitstellung entsprechender Ressourcen. [My university promotes the use of e-exams by providing appropriate resources.]
BI	01	Ich beabsichtige innerhalb der nächsten 2 Jahre E-Klausuren durchzuführen. [I intend to use e-exams within the next 2 years.]
	02	Ich werde E-Klausuren nur einsetzen, wenn ich dazu verpflichtet werde. [I will only use e-exams if I am obliged to do so.]*
	03	Ich werde E-Klausuren einsetzen, wenn ich entsprechende Unterstützung von Fachleuten erhalte. [I will use e-exams if I receive appropriate support from experts.]
	04	Ich werde E-Klausuren einsetzen, wenn die entsprechenden technischen Voraussetzungen gegeben sind. [I will use e-exams if the appropriate technical requirements are met.]
	05	Ich habe großes Interesse an der Nutzung von E-Klausuren [I am very interested in using e-exams.]
	06	Ich werde E-Klausuren zukünftig papierbasierten Klausuren vorziehen. [In the future, I will prefer e-exams to paper-based exams.]
	07	Ich werde künftig auf E-Klausuren, wenn irgend möglich, verzichten. [In the future, I will avoid e-exams if at all possible.]*
	08	Ich plane künftig E-Klausuren zu verwenden. [I plan to use e-exams in the future.]

* Inverted scoring. PU = Perceived usefulness, SN = Subjective norm; FC = Facilitating conditions; BI = Behavioral intention to use. Each item of these scales had a four-point Likert scale ranging from 1 *trifft gar nicht zu* [totally disagree] to 4 *trifft völlig zu* [totally agree].

## Appendix B

Table 7

**Table 7:** Results for the measurement model

	Group 1 (e-exams)			Group 2 (adaptive e-exams)		
Factor/Items	λ	*ω*	*AVE*	λ	*ω*	*AVE*
BI		.881	.705		.916	.683
BI01	.878			.954		
BI02	.790			.653		
BI03	.663			.819		
BI04	.774			.887		
BI05	929			.938		
BI06	.848			.776		
BI07	.861			.720		
BI08	.920			.818		
PU		.874	.624		.940	.713
PU01	.914			.908		
PU02	.870			.903		
PU03	.774			.803		
PU04	.757			.827		
PU05	.742			.806		
PU06	.670			.839		
PU07	.948			.948		
PU08	.669			.782		
PU09	.714			.762		
PE		.858	.545		.809	.519
PE01	.781			.733		
PE02	.784			.651		
PE03	.730			.703		
PE04	.727			.723		
PE05	.698			.641		
PE06	.646			.704		
PE07	.749			.742		
PE08	.701			.745		
PE09	.793			.734		
PE10	.753			.866		
PE11	.795			.689		
PE12	.687			.694		
PE13	.788			.749		
PE14	.685			.682		
CSE^a^		.853	.592		.835	.578
CSE05	.854			.891		
CSE06	.761			.734		
CSE07	.742			.721		
CSE10	.747			.709		
CSE11	.673			.678		
CSE12	.921			.914		
CSE13	.736			.705		
CSE14	.690			.692		
CA		.871	.595		.832	.587
CA01	.800			.902		
CA02	.773			.677		
CA03	.672			.694		
CA04	.745			.741		
CA05	.822			.852		
CA06	.749			.833		
CA07	.902			.718		
CA08	.684			.678		
SN		.717	.572		.722	.528
SN01	.699			.664		
SN02	.770			.708		
SN03	.789			.778		
SN04	.733			.723		
SN05	.787			.756		
FC		.873	.684		.902	.682
FC01	.862			.898		
FC02	.851			.847		
FC03	.644			.682		
FC04	.880			.873		
FC05	.821			.751		
FC06	.879			.883		

λ = Standardized factor loading; ω = Green and Yang’s (2009) variation of reliability coefficient ω; *AVE* = Average variance extracted; BI = Behavioral intention to use; PU = Perceived usefulness; PE = Prior experience; CSE = Computer self-efficacy; CA = Computer anxiety; SN = Subjective norm; FC = Facilitating conditions

^a^ Six items were removed due to limited variance

## Appendix C

Table 8

**Table 8:** Summary of hypotheses testing

		Group 1(e-exams)				Group 2 (adaptive e-exams)			
H	Path	β	*SE*(β)	*p*	As expected	β	*SE*(β)	*p*	As expected
1	PU➔BI	.793	0.024	≤ .001	Yes	.890	0.011	≤ .001	Yes
2	PE➔PU	.290	0.068	≤ .001	Yes	.356	0.073	≤ .001	Yes
3	CSE➔PE	.446	0.050	≤ .001	Yes	.456	0.057	≤ .001	Yes
4	CSE➔PU	.039	0.062	.532	No	-.080	0.070	.249	No
5	CSE➔BI	.030	0.028	.290	No	.006	0.023	.795	No
6	CA➔CSE	-.585	0.046	≤ .001	Yes	-.631	0.039	≤ .001	Yes
7	CA➔PE	-.042	0.068	.346	No	-.032	0.034	.150	No
8	SN➔PU	.531	0.030	≤ .001	Yes	.211	0.053	≤ .001	Yes
9	SN➔BI	.144	0.047	.002	Yes	.132	0.077	.015	Yes
10	FC➔BI	-.010	0.041	.808	No	-.045	0.029	.113	No

H = Hypothesis; β = Standardized path coefficients; *SE* = Standard error; BI = Behavioral intention to use; PU = Perceived usefulness; PE = Prior experience; CSE = Computer self-efficacy; CA = Computer anxiety; SN = Subjective norm; FC = Facilitating conditions
